# 1340. The Burden of Influenza and Rhinovirus Among Hospitalized Adults Post the COVID-19 Pandemic

**DOI:** 10.1093/ofid/ofab466.1532

**Published:** 2021-12-04

**Authors:** Olivia D Reese, Ashley Tippett, Laila Hussaini, Luis Salazar, Megan Taylor, Caroline Ciric, Laurel Bristow, Vikash Patel, Wensheng Li, Hui-mien Hsiao, Kathy Stephens, Theda Gibson, Ariel Kay, Andrew Cheng, David L Swerdlow, Robin Hubler, Ben Lopman, Christina A Rostad, Larry Anderson, Nadine Rouphael, Nadine Rouphael, Evan J Anderson

**Affiliations:** 1 Emory University School of Medicine, Atlanta, Georgia; 2 Pfizer, Inc, New York, NY; 3 Pfizer Inc, Collegeville, PA; 4 Rollins School of Public Health, Emory University, Atlanta, GA; 5 Emory University, Atlanta, GA; 6 Emory University, Atlanta VA Medical Center, Atlanta, Georgia

## Abstract

**Background:**

Acute respiratory tract infections (ARIs) are a significant cause of morbidity in adults. Influenza is associated with about 490,600 hospitalizations and 34,200 deaths in the US in the 2018-2019 season. The burden of rhinovirus among adults hospitalized with ARI is less well known. We compared the burden of influenza and rhinovirus from 2 consecutive winter respiratory viral seasons in hospitalized adults and healthy controls pre-COVID-19 and one season mid-COVID-19 to determine the impact of rhinovirus as a pathogen.

**Methods:**

From Oct 2018 to Apr 2021, prospective surveillance of adults ≥50 years old admitted with ARI or COPD/CHF exacerbations at any age was conducted at two Atlanta hospitals. Adults were eligible if they lived within an eight-county region around Atlanta and if their symptom duration was < 14 days. In the seasons from Oct 2018 to Mar 2020, asymptomatic adults ≥50 years old were enrolled as controls. Standard of care test results were included and those enrolled contributed nasopharyngeal swabs that were tested for respiratory pathogens using BioFire® FilmArray® Respiratory Viral Panel (RVP).

**Results:**

During the first two seasons, 1566 hospitalized adults were enrolled. Rhinovirus was detected in 7.5% (118) and influenza was detected in 7.7% (121). Rhinovirus was also detected in 2.2% of 466 healthy adult controls while influenza was detected in 0%. During Season 3, the peak of the COVID-19 pandemic, influenza declined to 0% of ARI hospitalizations. Rhinovirus also declined (p=0.01) but still accounted for 5.1% of all ARIs screened (Figure 1). Rhinovirus was detected at a greater rate in Season 3 than in asymptomatic controls in the first 2 seasons (p=0.008). In the first two seasons, Influenza was detected in 8.6% (24/276) of those admitted to the ICU. Rhinovirus was detected in 6.1% (17/276) of those admitted to the ICU but declined to 3.1% (8/258) in Season 3.

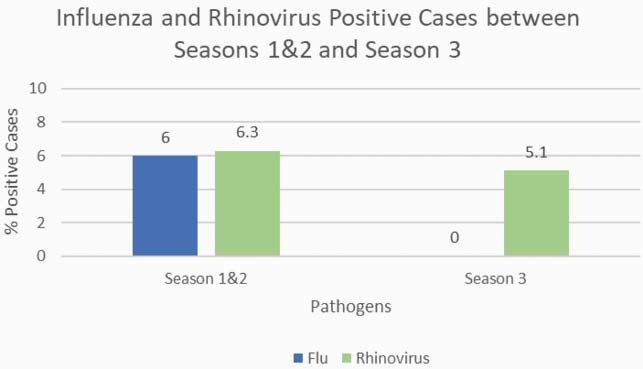

Figure 1. Percent Positive Cases of Influenza and Rhinovirus between Season 1&2 (hospitalized and healthy controls) vs Season 3 (hospitalized)

**Conclusion:**

Dramatic declines occurred in influenza in adults hospitalized with ARI, CHF, or COPD in Atlanta during the COVID-19 pandemic and with enhanced public health measures. Although rhinovirus declined during the COVID-19 pandemic, it continued to be identified at a rate higher than in historical controls. Additional data are needed to understand the role of rhinovirus in adult ARI, CHF, and COPD exacerbations.

**Disclosures:**

**David L. Swerdlow, MD**, **Pfizer Vaccines** (Employee) **Robin Hubler, MS**, **Pfizer Inc.** (Employee) **Christina A. Rostad, MD**, **BioFire Inc, GSK, MedImmune, Micron, Janssen, Merck, Moderna, Novavax, PaxVax, Pfizer, Regeneron, Sanofi-Pasteur.** (Grant/Research Support, Scientific Research Study Investigator, Research Grant or Support)**Meissa Vaccines** (Other Financial or Material Support, Co-inventor of patented RSV vaccine technology unrelated to this manuscript, which has been licensed to Meissa Vaccines, Inc.) **Larry Anderson, MD**, **ADVI** (Consultant)**Bavarian Nordic** (Consultant)**Novavax** (Consultant)**Phizer** (Grant/Research Support, Scientific Research Study Investigator)**Sciogen** (Research Grant or Support) **Nadine Rouphael, MD**, **pfizer, sanofi, lily, quidel, merck** (Grant/Research Support) **Nadine Rouphael, MD**, Lilly (Individual(s) Involved: Self): Emory Study PI, Grant/Research Support; Merck (Individual(s) Involved: Self): Emory study PI, Grant/Research Support; Pfizer: I conduct as co-PI the RSV PFIZER study at Emory, Research Grant; Pfizer (Individual(s) Involved: Self): Grant/Research Support, I conduct as co-PI the RSV PFIZER study at Emory; Quidel (Individual(s) Involved: Self): Emory Study PI, Grant/Research Support; Sanofi Pasteur (Individual(s) Involved: Self): Chair phase 3 COVID vaccine, Grant/Research Support **Evan J. Anderson, MD**, **GSK** (Scientific Research Study Investigator)**Janssen** (Consultant, Scientific Research Study Investigator, Advisor or Review Panel member)**Kentucky Bioprocessing, Inc** (Advisor or Review Panel member)**MedImmune** (Scientific Research Study Investigator)**Medscape** (Consultant)**Merck** (Scientific Research Study Investigator)**Micron** (Scientific Research Study Investigator)**PaxVax** (Scientific Research Study Investigator)**Pfizer** (Consultant, Grant/Research Support, Scientific Research Study Investigator)**Regeneron** (Scientific Research Study Investigator)**Sanofi Pasteur** (Consultant, Scientific Research Study Investigator)

